# Automatic Association of Chats and Video Tracks for Activity Learning and Recognition in Aerial Video Surveillance

**DOI:** 10.3390/s141019843

**Published:** 2014-10-22

**Authors:** Riad I. Hammoud, Cem S. Sahin, Erik P. Blasch, Bradley J. Rhodes, Tao Wang

**Affiliations:** 1 BAE Systems, Burlington, MA 01803, USA; E-Mails: cem.sahin@baesystems.com (C.S.S.); brad.rhodes@baesystems.com (B.J.R.); tao.wang@baesystems.com (T.W.); 2 Air Force Research Lab, Rome, NY 13441, USA; E-Mail: erik.blasch.1@us.af.mil

**Keywords:** activity recognition, FMV tracking, ATR, fusion, surveillance, pattern learning, features, registration, geo-registration

## Abstract

We describe two advanced video analysis techniques, including video-indexed by voice annotations (VIVA) and multi-media indexing and explorer (MINER). VIVA utilizes analyst call-outs (ACOs) in the form of chat messages (voice-to-text) to associate labels with video target tracks, to designate spatial-temporal activity boundaries and to augment video tracking in challenging scenarios. Challenging scenarios include low-resolution sensors, moving targets and target trajectories obscured by natural and man-made clutter. MINER includes: (1) a fusion of graphical track and text data using probabilistic methods; (2) an activity pattern learning framework to support querying an index of activities of interest (AOIs) and targets of interest (TOIs) by movement type and geolocation; and (3) a user interface to support streaming multi-intelligence data processing. We also present an activity pattern learning framework that uses the multi-source associated data as training to index a large archive of full-motion videos (FMV). VIVA and MINER examples are demonstrated for wide aerial/overhead imagery over common data sets affording an improvement in tracking from video data alone, leading to 84% detection with modest misdetection/false alarm results due to the complexity of the scenario. The novel use of ACOs and chat messages in video tracking paves the way for user interaction, correction and preparation of situation awareness reports.

## 1. Introduction

Streaming airborne wide area motion imagery (WAMI) and full-motion video (FMV) sensor collections afford online analysis for various surveillance applications, such as crowded traffic scene monitoring [1]. In a layered sensing framework, such sensors may be used to simultaneously observe a region of interest to provide complimentary capabilities, including improved resolution for target discrimination, identification and tracking [2]. Typically, forensic analysis, including pattern-of-life detection and activity/event recognition, is conducted off line due to huge volumes of imagery. This big data outpaces users' available time to watch all videos in searching for key activity patterns within the data. To aid users in detecting patterns in aerial imagery, robust and efficient computer vision, pattern analysis and data mining tools are highly desired [3].

### 1.1. Multi-Source Data and Problem Statement

**Figure 1.**
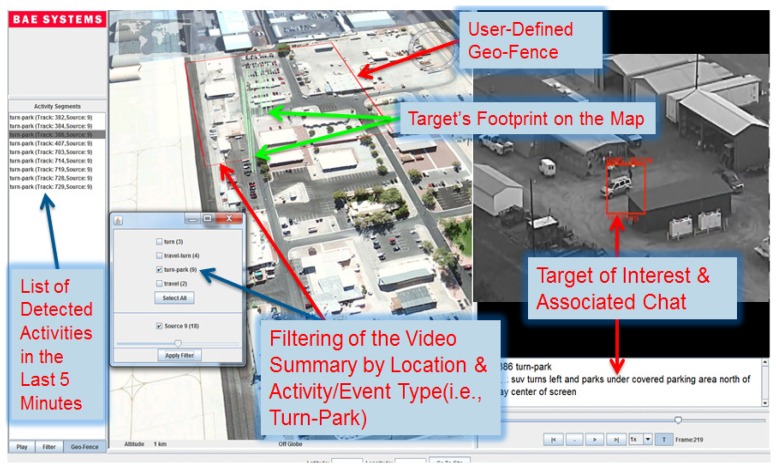
Illustration of the event-report visualization interface (multi-media indexing and explorer (MINER)) allowing users to visualize and query correlated chats, pattern of life and activity-labeled track segments.

For data collection and reporting, the aerial video is reviewed by humans (called hereafter reviewed FMV data), as the imagery is streamed down from an airborne platform. During a real-time FMV exploitation process, humans could call out significant AOIs, where a voice-to-text tool converts audible analyst call-outs (ACO) to text (see example in Figure 1), and a computer then saves the ACOs to storage disks along with the aerial imagery. Additional contextual information besides ACOs includes additional reviewers' (internal) chat, as well as discussions about the area of coverage of the overhead video from external sources. Together, the ACOs, internal discussions and external perspectives provide a collective set of “chat messages” [4].

However, these two data sources (chat messages and FMV) are not synchronized in time or in space. They are not recorded with corresponding time stamps. Furthermore, the called-out targets and activities are not marked in video frames with bounding boxes or with a start and an end of each activity. It is worth noting that the use of ACOs radically differs from a traditional video annotation paradigm that is typically done manually for training and/or benchmarking of computer vision algorithms. The incorporation of the user's ACO requires advances in automation, human-machine interaction and multimodal fusion. In addition, during the overhead imagery review process, there is no advanced equipment, such as an eye tracker [5] or touch screen employed to determine screen locations of the targets of interest (TOIs) corresponding to ACOs.

### 1.2. Paper Contributions

The ACO messages present a rich source of information allowing for a fast retrieval of activities and providing a summary of events over FMV. ACOs can provide a reference ground truth of the AOIs that occur in the reviewed FMV data. Hence, correlating these two data sources would produce four novel products: (1) a video summary of AOIs/TOIs allowing nonlinear browsing of video content; (2) annotated text-over-video media where only TOIs are highlighted with bounding boxes and synchronized with chat messages; (3) an activities index where linked events are grouped together; and (4) adaptive data play back allowing for user-selected filtering by geographic location. For instance, the end user may submit a query like this: pull out all video segments of activity types “turn then stop” near this house on the map (see Figure 1).

In this paper, we propose a multi-source probabilistic graph-based association framework to automatically: (1) identify TOIs corresponding to chat messages; (2) detect activity boundaries (*i.e.*, segmenting FMV tracks into semantic sub-tracks/segments); (3) learn activity patterns in low-level feature spaces using the reviewed FMV data; (4) index non-reviewed FMV data (*i.e.*, archived videos); as well as (5) assist FMV analysts with tools for fast querying and non-linear browsing of multi-source data.

Such an automatic linking process of multi-source data enhances data association by eliminating the tedious process of manually collecting and correlating the data. As a side benefit, pattern recognition typically requires training data for activity pattern learning; however, the chat messages provide a notional real-time training template. The need for activity training data has been well reported in the literature. For instance, [6] emphasizes the need to collect high-quality activity/event examples with minimal irrelevant pixels for the activity learning modules. Furthermore, during the manual annotation process, Oh *et al.* [6] define very specifically the start and end moments of activities to ensure proper learning on non-noisy data. Here, we demonstrate a paradigm shift in tracking and classification of imagery that does not require training data for real-world deployment of methods.

### 1.3. Paper Organization

Section 2 details related work. The following sections describe various components of our “video-indexed by voice annotations” (VIVA) system. Section 3 provides a video processing overview with extensions to our methods. Section 4 describes the mapping of a single FMV target track to multiple graphs of attributes. In Section 4.2, we describe our two-step algorithm to decompose a single track into semantic segments. Section 5 focuses on parsing of chat messages (or ACO) and their graphical representation. In Section 6, we present the multi-source graph-based association framework and the activity class assignment process. In Section 7, we briefly provide an overview of our approach for learning activity patterns from the reviewed FMV tracks (*i.e.*, training data) and querying the unlabeled FMV data. Sections 8 and 9 outline our multi-media indexing and explorer (MINER) interface and evaluate several scenarios to provide performance details of the proposed framework, respectively. We conclude the paper in Section 10.

## 2. Related Work

Visual activity recognition—the automatic process of recognizing semantic spatio-temporal target patterns, such as “person carrying” and “vehicle u-turn” from video data—has been an active research area in the computer vision community for many years [7]. Recently, the focus in the community has shifted toward recognizing activities/actions over large time scales, wide-area spatial resolutions [8] and multi-source multimodal frequencies in real-world operating conditions [9]. We assume here that a pattern is bounded by event changes, and target movement in between events is an “activity.” In such conditions, the major challenge arises from the large intra-class variations in activities/events, including variations in sensors (e.g., viewpoints, low resolution and scale), target (e.g., visual appearance, speed of motion) and environment (e.g., lighting condition, occlusion and clutter). The recognition of activities in overhead imagery poses many more challenges than from a fixed ground-level camera, mostly because of the imagery's low resolution. Additionally, the need for video stabilization creates noise, tracking and segmentation difficulties for activity recognition.

The key algorithmic steps in visual activity recognition techniques are: (1) extracting spatio-temporal interest point detectors and descriptors [10]; (2) performing clustering (e.g., K-means) in the feature space (e.g., histogram of oriented gradients (HOG), histogram of flow (HOF), histogram of spatio-temporal gradients (3D-STHOG) and 3D-SIFT) to form codebooks after principal component analysis (PCA)-based dimension reduction; and (3) labeling tracks using a bag-of-words approach [6,11]. We follow a similar process when it comes to learning activity patterns from the reviewed FMV tracks. That being said, we first perform multi-source data association to generate training data from the reviewed FMV tracks where FMV tracks are assigned activity labels.

Xiey *et al.* [12] proposed a method for discovering meaningful structures in video through unsupervised learning of temporal clusters and associating the structures with metadata. For a news-domain model, they presented a co-occurrence analysis among structures and observed that temporal models are indeed better at capturing the semantics than non-temporal clusters. Using data from digital TV news, Duygulu and Wactlar [13] proposed a framework to determine the correspondences between the video frames and associated text in order to annotate the video frames with more reliable labels and descriptions. The semantic labeling of videos enables a textual query to return more relevant corresponding images and enables an image-based query response to provide more meaningful descriptors (*i.e.*, content-based image retrieval). Our proposed activity recognition framework discovers meaningful activity structures (e.g., semantically-labeled events, activities, patterns) from overhead imagery over challenging scenarios in both reviewed and non-reviewed FMV data.

## 3. Multiple Target Tracking and Classification

Tracking multiple targets in aerial imagery requires first stabilizing the imagery and then detecting automatically any moving target. In this section, we briefly describe these techniques along with our automatic target recognition method.

### 3.1. Video Stabilization

Our frame-to-frame stabilization module aligns successive image frames to compensate for camera motion [14]. There are several steps involved in our two-frame registration process: (1) extracting interest points from the previous image that possess enough texture and contrast to distinguish them from one another; and (2) matching the 2D locations of these points between frames using a robust correspondence algorithm. Establishing correspondences consists of two stages: (1) use “guesses,” or putative matches, established by correlating regions around pairs of feature points across images; and (2) performing outlier rejection with random sample consensus (RANSAC) to remove bad guesses.

The VIVA stabilization algorithm runs in real time on “commercial, off-the-shelf” (COTS) hardware, and it was specifically designed to be robust against large motions between frames. The enhanced robustness against large motion changes is essential, since analog transmission of electro-optical/infrared (EO/IR) airborne data to the ground can be corrupted, frames can be dropped, time-delays long and can vary in sample rates. As long as the two frames being registered have greater than a 35% overlap, we are usually able to establish enough correspondences for reliable stabilization.

### 3.2. Target Detection and Tracking

**Figure 2.**
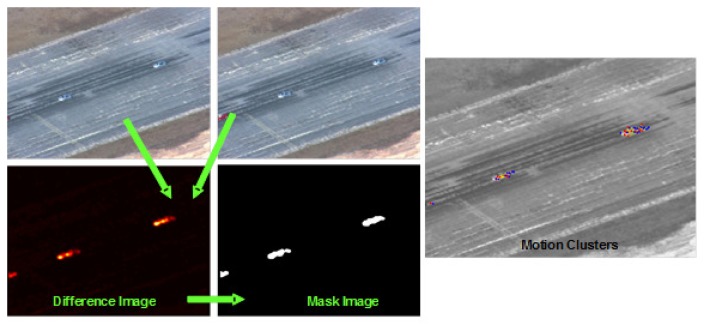
Video-indexed by voice annotations (VIVA)'s movement detection module. First, registered frames **(top left)** are differenced to produce a change detection image **(lower right).** That image is thresholded to detect changing pixels. Point correspondences within those detection pixels are established between the two frames and used to generate motion clusters **(right).**

**Figure 3.**
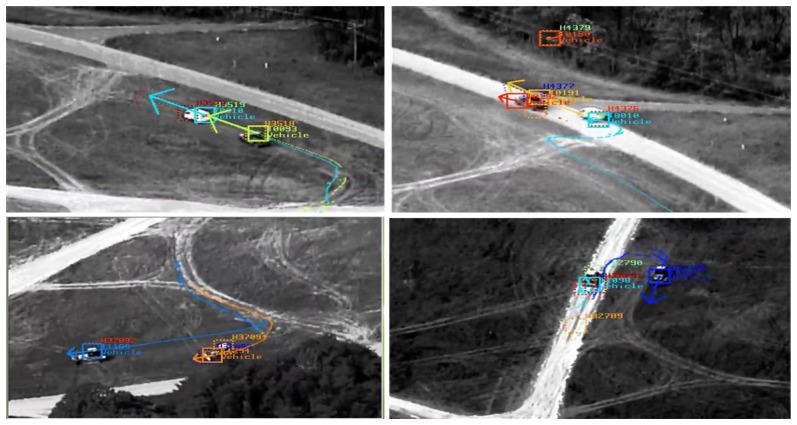
Example of track profiles of vehicles generated by COURSE using sample videos from the VIRATaerial dataset (ApHill) [6].

**Figure 4.**
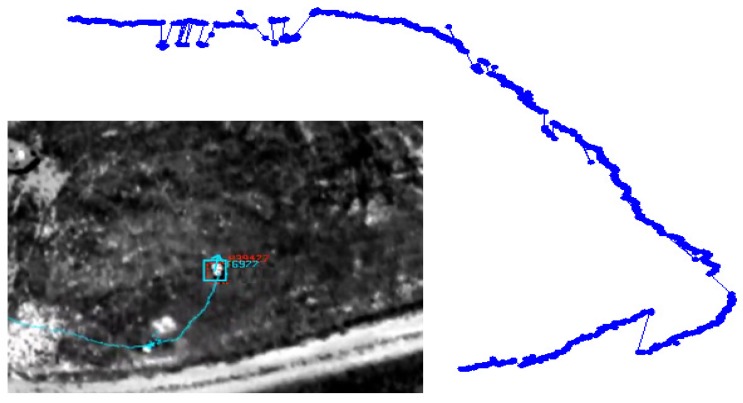
Illustration of a noisy tracking trajectory of a single dismount (from the ApHill VIRAT aerial dataset) generated by COURSE. The track is broken into several segments (*i.e.*, several tracking labels) due to quick changes in motion direction, cluttered background and multiple stop-and-move scenarios.

Our moving target tracking algorithm, cluster objects using recognized sequence of estimates (COURSE), makes few assumptions about the scene content, operates almost exclusively in the focal plane domain and exploits the spatial and temporal coherence of the video data. It consists of three processing steps. First, the frame-to-frame registration is used to find regions of the image where pixel intensities differ (this is done through frame differencing (see Figure 2)). Underlying frame differencing is the assumption that pixel intensity differences are due to objects that do not fit the global image motion model. Clearly, other effects, such as parallax, also cause false differences, but these false movers are filtered using subsequent motion analysis. Second, point features with a high pixel intensity difference are used to establish correspondences between other points in the previous frame, which produces a set of point-velocity pairs. Third, these point-velocity pairs are clustered into motion regions that we assume are due to individual targets. Regions that persist over time are reported as multiple target detections. The tracker provides two very important capabilities: (i) it removes false detections generated by the upstream target detection module; and (ii) extends detection associations beyond what can be accomplished by using only the image-based target detection module. COURSE achieves enhanced robustness by (i) removing isolated detections that are inconsistent with the presence of a moving object, and (ii) exploiting large time-event information to deal with brief interruptions caused by minor occlusions such as trees or passing cars. The COURSE tracker generates a mosaic tracking report (see Figures 3 and 4) to be used as input to our multi-source association framework.

### 3.3. Target Classification

In order to reduce the ambiguity in the multi-source association framework, we classify each target into “person,” “vehicle” or “others”. We employed support vector machines (SVM) with a radial basis function (RBF) to train models and classify unlabeled targets into these three categories. During training, we used a five-fold cross-validation process to find the best values for the radius of the RBF and the cost factor, which controls the importance of the training error with respect to the separation margin [15].

We extracted HOG and HOF to characterize the low resolution targets [10]. The HOG preserves some texture and local structure of the targets and is invariant to illumination changes. In contrast to HOG, the HOF features capture the motion information of the moving target. Once the supervised learning is completed, we classify every target track into one of the three categories using the majority vote from all individual frames of a track.

## 4. Multi-Graph Representation of a Single FMV Track

The multi-source association framework is based on a graph representation and matching of target tracks and chat messages. In this section, we describe how to build a graph-based model of a tracked target and how to divide long and informative tracks into semantic track segments and, hence, represent a single track with multiple graphs.

### 4.1. Mapping Tracks to Graphs

**Figure 5.**
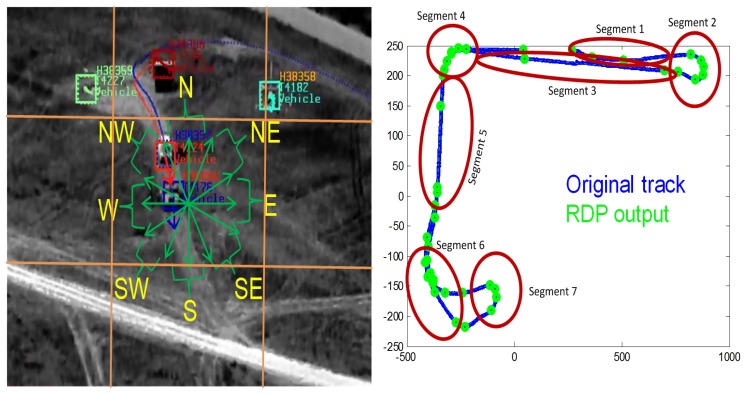
Illustration of our assignment of tracking states into (a) direction and location zones (e.g., south-east direction, top-left screen zone, *etc.*) and (b) semantic segments based on changes in direction and speed using RDP.

Each target track is cast by a combination of graphs where nodes represent the targets' attributes and edges characterize the relationship between nodes. We divided attributes into common and uncommon based on their saliency over the lifetime of a target track. For instance, the color and shape of a vehicle remain unchanged, while direction and spatial location vary over time (*t*). The TOIs are classified into “vehicle” *vs.* “human” (*i.e.*, actor attribute) based on motion, blob size and shape. The shape attribute is divided into “car” *vs.* support utility vehicle “SUV” *vs.* “truck” for vehicle, and “adult” *vs.* “child” for human actor/dismount [16]. Each actor is characterized with a unique color attribute (e.g., black truck, human with red-shirt, *etc.*) and a spatial location (*i.e.*, *xy_s_* position on the screen and latitude/longitude on the geographic map). The location is mapped into gross zones (see Figure 5a) on the screen to match with gross locations in the chat messages. We divided the video frame into a 3 × 3 grid (center screen, top left, *etc*). The direction attribute is derived from the velocity vectors (*V_x_*(*t*), *V_y_*(*t*)) at time *t*, such that $\theta (t)=\text{arctan}(\frac{V_{y}(t)}{V_{x}(t)})$, which, in turn, is mapped to a geographical direction using the gross divisions of directions, as shown in Figure 5a. In order to reduce noise in the mapping of *θ* and *xy_s_* to gross direction and location zones, we applied a sliding window to smooth these values over time. The last attribute is mobility, which specifies whether the target is moving or stationary (*m_t_*).

### 4.2. Dividing Tracks into Semantic Segments

When a track exhibits major changes in uncommon attributes, especially in direction, location and speed, it becomes necessary to break it down into multiple semantic segments, which results in multiple graphs in the association framework. Track segmentation into graphs is needed when multiple chats correspond to a single track generated by our video tracker. Figure 5b shows three minutes of a tracked vehicle moving toward the east, making a u-turn then moving toward the west. We apply a two-step algorithm to break down tracks into semantic segments:

(1) Smooth the tracking locations (*xy_s_*) using the Ramer-Douglas-Peucker (RDP) algorithm [17]. RDP will produce a short list of un-noisy position points (*XY_s_*) (displayed as green points in Figure 5b).
(2) Detect directional changes computed from *XY_s_*(*t*) points. The beginning of a new semantic track segment is marked when a peak is detected. The end of a new semantic segment is flagged when the second derivative of *XY_s_*(*t*) is near zero. Figure 5b illustrates the results of this step, where seven segments were detected.

## 5. Parsing and Graph Representation of Chats

In our data collection setup, the chat messages follow the following format for a target of type vehicle [18]:
At <time> <quantity> <color> <vehicle><activity> <direction> <location>where:<time> = 0000Z − 2359Z<activity> = (travel | u–turn …)<direction> = (north | south …)<location> = screen (middle | left …)<color> = (red | black …)<shape> = (truck | car …)

Basic search for keywords in a chat message is employed to extract relevant information, such as “activity type,” “direction,” and “location.” In our dataset, we have nine activities (vehicle turn, u-turn, human walking, running, *etc.*; see Section 9), eight direction zones (north, south, *etc.*) and nine location zones (middle, top-left screen zone, *etc*; see Figure 5).

These chat messages represent an analyst calling out activities in the FMV, intra-viewer discussions or other related external discussions. In turn, a chat message is represented as a graph of attributes. However, more elaborated information extraction (IE) from a chat message (*i.e.*, micro-text) or a document (e.g., using Sphynx or Apache NLP) as an automated approach [19–22] could be employed to handle misspelled words and larger dictionaries.

**Figure 6.**
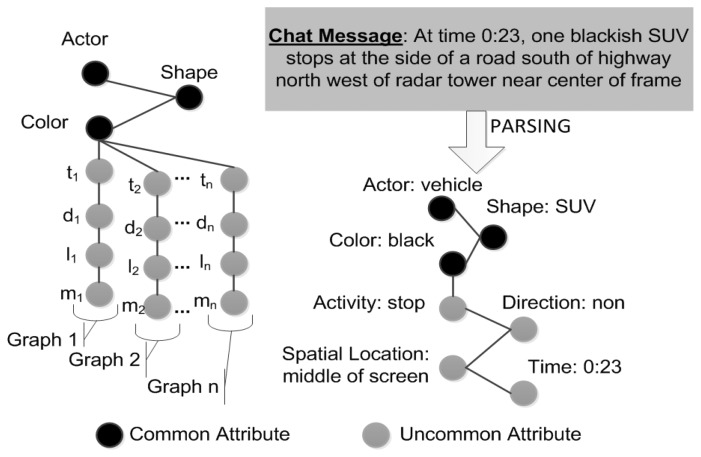
Example of representation of a video track (a) and a chat message (b) as graphs.

Figure 6b illustrates a chat message decomposed into multimodal attributes. An example can come from any modality (e.g., video, text, radar, *etc.*), so the goal is to decompose the data into these meaningful parts [4].

## 6. Multi-Source Graph Association and Activity Class Assignment

A mission goal includes allowing the image processing method to answer a user-defined query. The user calling out significant activities in the image would desire an automated processor to match the target being called out to that of a TOI. Within an image in a video stream, there could be many movers, targets and events happening. The system must choose the TOI among several tracked objects in the imagery that corresponds to a meaningful content (attributes) in the chat message by a user. Because users review FMV tracks from streaming airborne video, the callouts flag AOIs. Association between reviewed FMV tracks and chat messages can be achieved by performing probabilistic matching between graphs from both data sources. It is important to note that, as explained in the Introduction, a chat message is the only source to describe the true activity of the TOI. By performing multi-source graph-based association, the true activity of the TOI is mapped to a corresponding track segment from FMV.

The multi-source multimodal association framework consists of the following stages:

1. In a given time interval, [*t* − *T*, *t* + *T*] (with *t* the time stamp from a chat message and *T* : a predefined time window to search for the tracked objects), the chat message and all video tracks are extracted from the data sets.
2. Graph representations of video tracks and chat messages are generated as explained in Sections 4 and 5.
3. Graph matching uses a probabilistic distance measure (see Equation (1)) of ensemble similarity between a chat message (*j*) and track segment (*i*). There are three main reasons to use a probabilistic distance metric: (i) to associate the graphs even if there are missing attributes; (ii) to reduce the effects of errors coming from the video processor and chat messages (e.g., a user may assign a vehicle color as black, while a tracked object from the video processor might be marked as gray); and (iii) to impute the weights of attributes based on the quality of videos. The associated graphs with the highest likelihoods are assigned as a match.
  (1) $$P(T_{i}|C_{j},c_{i})=w_{a}P_{a}+w_{s}P_{s}+w_{t}P_{t}+w_{cl}P_{cl}+w_{d}P_{d}+w_{l}P_{l}+w_{cn}P_{cn}+w_{m}Pm$$
  where *w_a_*, *w_s_*, *w_t_*, *w_cl_*, *w_d_*, *w_l_*, *w_cn_* and *w_m_* are the user-defined weights of attributes for actor, shape, time, color, direction, spatial location, tracking confidence and target mobility, respectively. *P_a_*, *P_s_*, *P_t_*, *P_cl_*, *P_d_*, *P_l_*, and *P_m_* represent the probabilities of corresponding attributes, and *P_cn_* is the track confidence value generated by COURSE.

**Figure 7.**
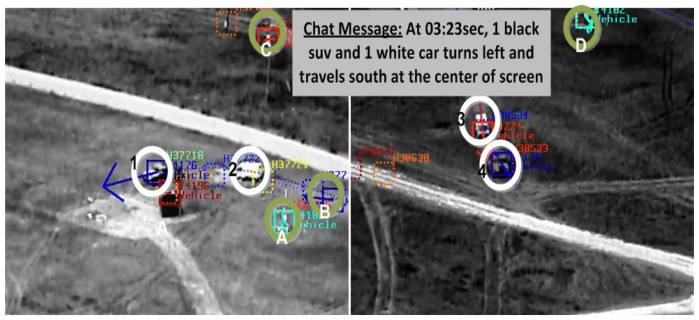
Successful identifications of AOIs/TOIs in exemplar clips from the ApHill VIRAT aerial dataset using our multi-source association framework. (**a**) and (**b**) show multiple vehicle tracks and a single chat message being called-out; the tracks in white circles (1, 2, 3 and 4) were highly matched with the chat message graphs, while targets in green circles (*A*, *B*, *C* and *D*) scored low matching probabilities.

An illustrative result of this framework is shown in Figure 7. This framework handles one-to-one, one-to-*N*, and *N*-to-*M* association cases. Furthermore, this framework not only marks the TOI, but also the rendering of activities. Using labeled track profiles, the boundaries of each activity are determined by using the process described in Section 4.2. For example, after labeling each track segment by associating chat messages, track Segments 1, 3 and 5 are marked as travel, Segments 2 and 7 are u-turn and track Segments 4 and 6 are labeled as turn in Figure 5b.

## 7. Learning Activity Patterns from Multi-Source Associated Data

The chat messages provide the pseudo ground truth of the AOIs occurring in the reviewed FMV video (see Section 6). These correlated data also serve as training data for activity pattern learning in aerial imagery. Here, we employ BAE Systems' Multi-intelligence Activity Pattern Learning and Exploitation (MAPLE) tool, which uses the hyper-elliptical learning and matching (HELM) unsupervised clustering algorithm [23] to learn activity patterns. This is done through extracting features from each labeled track segment, clustering features in each activity space and finally representing each track by a sequence of clusters (*i.e.*, chain code). In terms of features, we used simple descriptors for vehicles, including speed, heading relative to segment start and a position eigenvalue ratio. By measuring the change relative to a fixed starting value, rather than the instantaneous change, the heading feature is robust to variations in how quickly the targets turns from its initial course. The position eigenvalue ratio is a measure of the mobility of the target, which is the ratio of eigenvalues calculated from the target's position within a short time duration. As for people tracking, we compute the histogram of motion flow and neighboring intensity variance, which describes the extent to which the target is moving toward or away from potential interaction sites.

The goal of the learning process is to be able to match an unlabeled track (*i.e.*, without chat) to the indexed pattern of life trajectories over large amounts of non-reviewed FMV data. First, we use HELM to classify each instance of a new track to one of the clusters of the index. Second, we use temporal gradient matching distance to obtain matches between the representation of the new track and the indexed learned patterns. The similarity score between a new track *j* and an index *i* is denned as follows:

(2) $$\sigma_{ij}=\frac{t_{ij}+c_{ij}}{2}$$

where *t_ij_* represents the similarity metric, which considers only the common clusters, and *c_ij_* is the similarity score of temporal gradient for the cluster sequence.

## 8. Event/Activity Report Visualization and Querying by Activity Type and Geolocation

The VIVA and MINER framework produces three useful products for the end users to visualize in the same interface (see Figure 1): (1) a video summary of AOIs allowing nonlinear browsing of video content; (2) text-over-video media where only TOIs are highlighted with bounding boxes and synchronized with chat messages, which describe their activities; and (3) an index of activities. The benefit of the compiled index of videos is that a user (or machine) could find related content over a geographic location and text query. For instance, the end-user may submit a query like this: pull-out all video segments of activity types “turn then stop” near this house with specific latitude and longitude coordinates. We converted each track to geo-tracks using both meta-data and reference imagery. If the AOI is detected in archived video using the activity classification framework presented above, the chat panel in our MINER interface shows the automatically generated description (*i.e.*, target category and activity type).

## 9. Experimental Results

To validate the proposed framework, we used our own dataset consisting of EO/IRairborne videos (60 min long) and 100 chat messages. The activity list is limited to vehicle travel, stop, turn, u-turn, maintain-distance, accelerate and decelerate and human walking and running. The VIVA video tracker generated about 8000 different tracks. The percentage of false tracking is about 15 percent and could be reduced through fusion of both EO and IR [24]. This is mainly due to camera zoom-ins and zoom-outs, which happen frequently when the camera focuses on TOIs to enhance resolution.

Each object was automatically classified into one of the three categories: “person,” “vehicle” or “others.” These models were trained offline using 34, 681 instances/frames of 514 tracked targets. We obtained 72.16% and 76.99% correct classification in HOF and HOG spaces, respectively. After majority voting, the track-wise classification accuracy increased to 86.58% and 92.61% in HOF and HOG, respectively. The biggest confusion is between humans and “others”, due to the low resolution of human targets. Most of the false targets that should be classified as “others” are due to noisy registration, platform motion and inaccuracy of the tracking bounding boxes, which often included parts of the background.

Specific low-level features (see Section 4.1) were computed prior to running the proposed multi-source multimodal association framework. Table 1 summarizes the results of the association framework. Correct associations are marked when the tracks or sub-tracks (*i.e.*, semantic segments) in the overhead imagery are associated with their corresponding chat messages. A false association is flagged when the chat message is linked to the wrong target or semantic segment (see Section 4.2). This could occur when multiple targets are moving in the same area at the same time and in the same direction. A miss is defined as a chat message without an associated target in the overhead imagery. On this data set, we scored 83.77% correct association, 6.39% misses association and 9.84% wrong association (*i.e.*, false alarms). During these experiments, we set the time window in which to perform the multi-source associations to 15 seconds. Making this window shorter leads to less false alarms, but also a higher miss rate. Furthermore, we only used the target's direction, location and speed as attributes, which do not include other useful content to reduce false alarms.

The association framework for activity recognition handles complex scenarios with multiple tracked objects. Figure 7a and b shows eight different tracks (different track labels) of six moving objects and a single chat message called out within the same time window. The chat message is parsed automatically into four different graphs, which are matched to all ten graphs representing the video tracks. The additional two video graphs (initially, we got eight tracks) came out from the splitting process of a single track into semantic segments (or sub-tracks, as described in Section 4.2), due to changes in vehicle direction while traveling. The VIVA framework associated the four chat graphs to the correct four FMV semantic tracks due to strong matches between common attributes. Our approach was also challenged by broken tracks (e.g., the case of a dismount/TOI with three different tracking labels in Figure 4). In spite the fact that the same TOI is represented by three consecutive tracks, VIVA provides correct associations with event boundaries (*i.e.*, shorter and semantic track segments). Thus, it is robust to scenario variations.

These preliminary results are very promising. Both the direction and the location attributes play an important role in the association of chat messages to tracks. The list of potential matches is reduced drastically using these attributes. Nevertheless, in order to make a one-to-one association, additional attributes, such as shape, color and size, and spatial relationships, such as a target near an identifiable landmark in the scene, would be very helpful to resolve association ambiguities. Due to the chat description, the extracted target's direction and location are cast to gross zones (*i.e.*, middle screen region, northeast direction, *etc.*) rather than fine ranges, causing ambiguities in the association. Extracting buildings from available imagery [25] would greatly benefit the association, because the chats refer to such attributes when describing activities involving human-object interactions.

**Figure 8.**
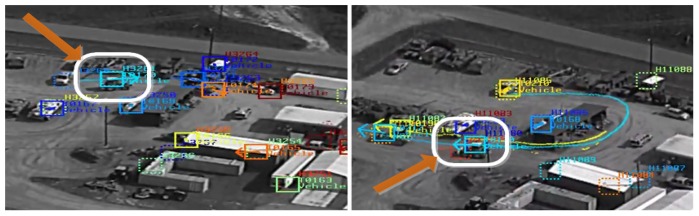
Illustration of an exemplar target track (from the ApHill VIRAT aerial dataset) being matched to the proper activity pattern model (a u-turn in this example) learned using the training data generated by the proposed multi-source association approach.

We used the multi-source associated data to learn activity patterns and then to index non-reviewed data (see Section 7). We tested this framework on 54 new track segments/activities and obtained 79.6% correct activity label assignment (see Table 2). Figure 8 shows a track segment from an unlabeled video correctly matched to a u-turn pattern model with a highest matching score *σ_q,uTurn_* ≈ 1.0 compared to other models. It is worth mentioning that the automatically generated training data from the multi-source association framework is not noise-free with a near 10% false classification (see Table 1). Add to that the errors that come from the automatic target classification and event boundary detection. Further, misclassified targets as vehicles add noise to the pattern learning process. It is necessary to have the human in the loop to correct the automatically generated training data prior to the activity pattern learning process. Additionally, having more training data will ensure building reliable pattern activity models in challenging operating conditions with enough intra-class variations using high dimensional activity descriptors over a larger activity list.

## 10. Conclusions

In this paper, we developed a novel concept for the graphical fusion of video and text data to enhance activity analysis from aerial imagery. We detailed the various components, including the VIVA association framework, the COURSE tracker, the MAPLE learning tool and the MINER visualization interface. Given the exemplar proof of concept, we highlighted the benefits for a user in reviewing, annotating and reporting on video content. Future work will explore the metrics and associations used to increase robustness and to reduce false alarms. However, it is noted that the end user can check the final results presented in our MINER interface to remove false alarms and effortlessly generate mission reports.

## Figures and Tables

**Table 1.** Qualitative assessment of the multi-graph association and activity class assignment framework.

**Detection**	**Miss**	**False**
83.77%	6.39%	9.84%

**Table 2.** Qualitative assessment of the classification of unlabeled tracks into the seven learned activity patterns.

**Correct**	**False**
79.6%	20.4%
